# Healthcare Experiences of LGBTQ+ Elders: Two Focus Groups

**DOI:** 10.1093/geroni/igaa057.257

**Published:** 2020-12-16

**Authors:** Kierra Foley, Nancy Hodgson, José Bauermeister

**Affiliations:** 1 School of Nursing, University of Pennsylvania, Philadelphia, Pennsylvania, United States; 2 University of Pennsylvania, Cherry Hill, New Jersey, United States

## Abstract

LGBTQ+ elders experience significantly higher rates of disability, poor general health, and behavioral health problems than heterosexual and cisgender older adults; additionally, LGBTQ+ elders are less likely to seek medical attention or have a primary care provider than other older adults. In previous qualitative studies, LGBTQ+ elders have reported difficulties navigating healthcare systems due to provider incompetence in treating this population, financial barriers, systemic homophobia/transphobia, and high disease burdens. However, few qualitative studies have been conducted with this population from a nursing perspective, and little is known about the role of nurses in the health and wellness of LGBTQ+ elders. Nurses are uniquely positioned to help address both the physiological and psychosocial needs of older LGBTQ+ adults, and garnering an understanding of these needs directly from this community can help better direct future research. Accordingly, two focus groups were conducted with LGBTQ+ elders at different queer community centers in Philadelphia by a nurse scientist. This community engaged research aims to learn more about how nurses can serve this population through identifying their priorities. Through thematic analysis, it was found that LGBTQ+ elders reported being concerned about housing access, discrimination from healthcare providers, and challenges in accessing mental health care. Additionally, the researchers’ hypotheses were triangulated with the participants’ responses to demonstrate the utility of continued community engagement in addressing specific population needs.

